# Recombination produces coherent bacterial species clusters in both core and accessory genomes

**DOI:** 10.1099/mgen.0.000038

**Published:** 2015-11-05

**Authors:** Pekka Marttinen, Nicholas J. Croucher, Michael U. Gutmann, Jukka Corander, William P. Hanage

**Affiliations:** ^1^​ Aalto University, Espoo, Finland; ^2^​ Imperial College, London, UK; ^3^​ University of Helsinki, Helsinki, Finland; ^4^​ Center for Communicable Disease Dynamics, Harvard School of Public Health, Boston, MA, USA

**Keywords:** computational modeling, core/accessory genome, evolution, recombination, speciation

## Abstract

**Background:**

Population samples show bacterial genomes can be divided into a core of ubiquitous genes and accessory genes that are present in a fraction of isolates. The ecological significance of this variation in gene content remains unclear. However, microbiologists agree that a bacterial species should be ‘genomically coherent’, even though there is no consensus on how this should be determined.

**Results:**

We use a parsimonious model combining diversification in both the core and accessory genome, including mutation, homologous recombination (HR) and horizontal gene transfer (HGT) introducing new loci, to produce a population of interacting clusters of strains with varying genome content. New loci introduced by HGT may then be transferred on by HR. The model fits well to a systematic population sample of 616 pneumococcal genomes, capturing the major features of the population structure with parameter values that agree well with empirical estimates.

**Conclusions:**

The model does not include explicit selection on individual genes, suggesting that crude comparisons of gene content may be a poor predictor of ecological function. We identify a clearly divergent subpopulation of pneumococci that are inconsistent with the model and may be considered genomically incoherent with the rest of the population. These strains have a distinct disease tropism and may be rationally defined as a separate species. We also find deviations from the model that may be explained by recent population bottlenecks or spatial structure.

## Data Summary

1 Supplementary Animations have been deposited in Figshare: http://figshare.com/s/6471c982669011e58c4806ec4b8d1f61
2 r code to run the model has been deposited in Figshare: http://figshare.com/s/c70dd5e0669011e59ff906ec4bbcf141

## Impact Statement

Bacterial species should be ‘genomically coherent’, but what this means is unclear due to the horizontal gene transfer that they exhibit. We fit a simulation of diversification in the core and accessory genome, including horizontal transfer, to a sample of >600 pneumococcal genomes, capturing the major features of the data and providing estimates of key parameters highly consistent with independent empirical measurements. The model predicts the surprising observation that all but one of the major strain clusters in the data are equidistant from each other as measured in terms of either core or accessory genome divergence – a feature that we show can be produced by biologically plausible recombination rates. Notably, the model is neutral with regard to the fitness of the different gene combinations that make up each genome. Deviations from model prediction indicate a departure from neutral expectations worthy of further investigation: strains that are more divergent than expected may be defined as a distinct species, suggesting a rational basis for the definition of a genomically coherent species. Strains that are more closely related may reflect short-term selective and epidemiological processes.

## Introduction

Bacterial diversity can be described in terms of the alleles of core genes common to all strains and the additional accessory genes present in a subset of strains. For example, as little as 11 % of all *Escherichia coli* genes described are present in all strains of the species (Perna *et al.*, 2001; Touchon *et al.*, 2009), and the concepts of the ‘core’ and ‘pan’ genomes are now commonplace. Variation in gene content is often assumed to be selective, reflecting different ecological specialization, but this has rarely been formally tested (Baltrus, 2013) and evidence exists that the selective consequences of horizontal gene transfer (HGT) may be surprisingly small (Knöppel *et al.*, 2014). The profusion of large population-based studies of individual pathogens presents us with an opportunity to test different models of diversification, explicitly examining the expected core and accessory genome distribution.

**Fig. 1:**
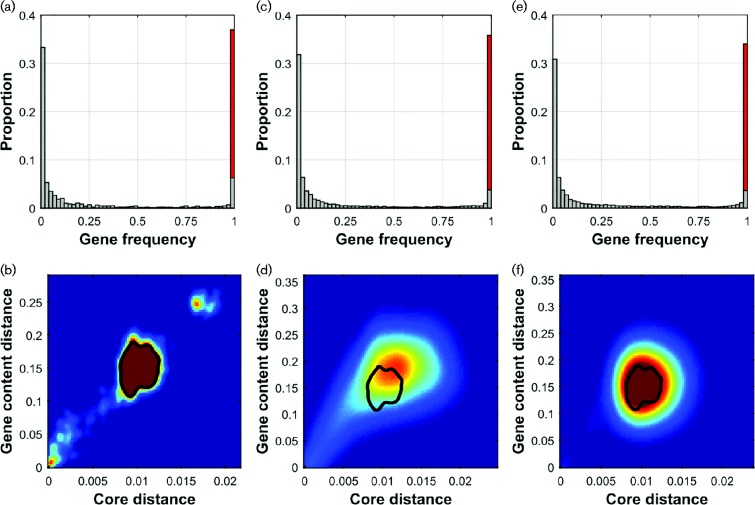
Gene frequency histograms (a, c, e) and strain distance distributions (b, d, f). The frequency histograms (a, c, e) show the number of very rare or common genes is much larger than the number of genes at intermediate frequencies; the red column represents the core genome (the overlapping grey bar represents frequencies *f* with 0.98 < *f* < 1). The distance distributions (b, d, f), obtained by averaging over the whole simulation after discarding initial samples, are based on pairwise comparisons of strains, showing the core genome (Hamming) distance on the *x*-axis and the gene content (Jaccard) distance on the *y*-axis (see Methods). A contour line encompassing the mode in the real data is shown in the simulated distributions for easier comparison. The columns show results in the real data (a, b), in the model with learned parameter values (c, d) and in the model with between-strain recombination increased by a factor of 10 (e, f).

Models of diversification in the core genome point to the vital role of homologous recombination (HR) in forming clusters of related strains and maintaining population structure (Fraser *et al.*, 2007, 2009; Doroghazi & Buckley, 2011). However, these models do not account for recombination events affecting the gene content. On the other hand, several models have successfully provided insight into how gene content evolves to produce the characteristic U-shaped histogram of gene frequencies observed at multiple levels of taxonomy (an example is shown in Fig. 1a) (Baumdicker *et al.*, 2012; Collins & Higgs, 2012; Haegeman & Weitz, 2012; Lobkovsky *et al.*, 2013). Many extensions also exist: expanding population (Baumdicker *et al.*, 2012), genes with different fitnesses (Lobkovsky *et al.*, 2013), and multiple gene categories with different deletion/acquisition rates (Collins & Higgs, 2012; Haegeman & Weitz, 2012). These models have included rates of acquisition and loss of genes, but have not modelled the divergence of the core simultaneously with that of the accessory genome nor investigated the potential for gene exchange by HR. The recent emergence of population genomics has produced datasets of hundreds or thousands of genomes from the same species, sampled in a systematic fashion (Croucher *et al.*, 2013; Chewapreecha *et al.*, 2014). Here, we present a model that includes both core genome and gene content variation, and use it to examine a well-characterized collection of 616 *Streptococcus pneumoniae* genome sequences (Croucher *et al.*, 2013).

The joint distribution of core genome and gene content divergence in the data shows that gene content, measured here in terms of clusters of orthologous groups (Tatusov *et al.*, 1997), diverges approximately linearly with core genome sequence (Croucher *et al.*, 2014) (Fig. 1b). The dominant feature in the distribution is the concentration of the majority (∼86 %) of the distances within a small, clearly delineated region. This mode results from the fact that all but one of the 15 major sequence clusters detected in the population are approximately equally distant from each other by both metrics (Croucher *et al.*, 2013). Another small mode near the origin corresponds to distances between very closely related strains and the small mode in the top-right corner represents strains in the single more divergent cluster; these strains have previously been characterized as ‘atypical pneumococci’ (Croucher *et al.*, 2014).

## Methods

### Model

Previous models have considered the observed diversity in the core genome of loci present in all strains (Fraser *et al.*, 2007, 2009). We extend this to include the accessory genome, with parameters governing the gain and loss of genes. Here, we present an overview of our approach; a detailed description of the model and the model fitting algorithm are provided in the supplementary text, Figs S1–S5, and Tables S1 and S2. Briefly, we simulate a population of sequences according to the Wright–Fisher model by sampling with replacement from the previous generation, with the following events possible at each generation: gene introduction, gene deletion, HR (replacing the recipient allele with the donor allele), HGT between two strains (altering the genome content in the recipient) and mutation. Our model is parsimonious, with just five free parameters representing rates of the different events, and is neutral with respect to the success of individual genes or lineages, and the resulting association between the core and accessory loci. A small multiplicative fitness penalty (using a factor of 0.99; values in the range from 0.95 to 0.999 produced similar results; see Figs S14–S16 for sensitivity analyses) is imposed for each gene exceeding a prespecified genome size, to prevent the genome growing without limit. Recombination events are accepted with a probability that decreases exponentially with increasing sequence divergence, reflecting a log-linear decline in the frequency of recombination with the divergence of donor and recipient sequences, as observed in empirical studies (Vulić *et al.*, 1997; Majewski *et al.*, 2000; Zawadzki *et al.*, 1995). To reduce computational complexity further, we use a low-dimensional representation for the gene sequences and approximate the real distances with Monte Carlo simulation.

### Model fitting

A normal maximum-likelihood approach to model fitting is computationally infeasible, so we use simulation-based inference, and match summary statistics between the simulated and real data; this resembles the simulated method of moments (McFadden, 1989; Pakes & Pollard, 1989; Gouriéroux & Monfort, 1997; Wood, 2010). To determine the parameter value maximizing the similarity between simulated and real data, we model the overall similarity score over a range of plausible parameter values by non-parametric regression (Rasmussen, 2006; Gutmann & Corander, 2015). The model fitting procedure involves a subjective decision on selecting data summaries to use when comparing between real and simulated data. We used one multivariate and three scalar summaries, all of which varied systematically in the simulation, allowing unambiguous identification of the model parameters. The multivariate summary was the U-shaped gene frequency histogram (Fig. 1a), which was highly informative about gene deletion and introduction rates (Fig. S6). For determining the HGT and HR rates, we defined two additional data summaries, termed here as the ‘clonality score’ and the ‘linkage score’, respectively. These measure the randomness of the distribution of the accessory genes in the population and the correlation between core loci, with high rates resulting in low scores (Figs S4 and S5). The slope of the distance distribution (Fig. 1b) was used as the last statistic informative about mutation rate. Namely, high mutation rate stretched the distribution along the *x*-axis, resulting in a more gradual slope.

### Distance metrics

The Hamming distance between two strains, used to measure the core genome divergence, measures the proportion of differing sites in the core genome alignment. The Jaccard distance, used to measure the gene content divergence, equals the number of genes present in one and absent in the other strain, divided by the total number of genes present in either one of the strains.

### Data

For simplicity, we use a term ‘gene’ to refer to a cluster of orthologous groups throughout this paper. Core gene alignments, cluster annotation of the strains, the gene presence–absence matrix and a phylogenetic tree have been described previously (Croucher *et al.*, 2013). As an additional data cleaning step, we removed all genes whose alignment length was < 265 bp, which corresponded to the 0.05th quantile of the lengths of the alignments of the core genes. This step was added to increase confidence in the genes detected. This left us with 2692 accessory genes and 1191 core genes in the 616 pneumococcal isolates. The detailed genomic analysis estimates for gene introduction and deletion rates in the real data, provided in Table 1, were obtained by estimating maximum-likelihood reconstructions of the genes along the fixed phylogeny, using an r function ace from package ape (Paradis *et al.*, 2004). The number of substitutions introduced by recombinations versus mutations, *r*/*m*, was computed as the mean over estimates reported for the sequence clusters (Croucher *et al.*, 2013).

**Table 1:** Estimates for two parameters: *r*/*m* (the number of substitutions introduced by recombinations versus mutations) and the ratio of gene introduction/deletion rates The second column reports the estimate from the model and the third column an estimate from a detailed genomic analysis (see Methods).

Parameter	Model estimate	Genomic analysis
*r*/*m*	8.0	11.3
Gene introduction/deletion	1.3	1.4

## Results and Discussion

Our fitted model predicts a stationary mode in the distance distribution, in the same location as in the real data, and increasing the recombination rate does not alter its location (Fig. 1). Thus, the mode appears to represent a limit for divergence in the population similar to what has previously been reported from gene sequence models (Fraser *et al.*, 2007), but, strikingly, we see a similar limit in the divergence of gene content. Note that the model was fit without assuming the mode, using metrics in the model fitting process that were independent of the mode. Altering the recombination rate has a major impact on dynamics. Whilst the position of the mode is consistent when averaged over time, it can move markedly over short timescales and separate into multiple clusters (see Animations S1–S3). With extremely low recombination rates, the observed mode does not emerge and the model output is merely distinct groups of closely related strains drifting rapidly apart from each other. After the mode emerges, increasing recombination within the population (i.e. the HR and HGT rates, see Model), whilst maintaining other parameters in their fitted values, does not change its location but rather stabilizes it. This indicates the impact of recombination on the population structure as measured here saturates when the distribution of alleles/genes between strain clusters is close to random, which is the required condition for the mode to emerge. The saturation can be seen in the levelling of the scores used in model fitting (Figs S4 and S5). For example, when two loci have become relatively uncorrelated due to recombination, further recombination has little impact.

Fitted values of the five parameters are shown in Table S1. In addition to the raw values, we recorded information of all events during the simulation, from which we computed the total number of substitutions introduced by HR and mutation, and the total number of gene introductions (caused either by an introduction of a new gene into the population or a within population gene transfer) and the overall number of gene losses (caused either by deletions or within population gene transfers). The resulting estimates of the ratio of recombination to mutation and the acquisition and loss of novel loci reported in Table 1 broadly recapture estimates from previous work analysing sets of whole-genome alignments (Croucher *et al.*, 2013).

**Fig. 2:**
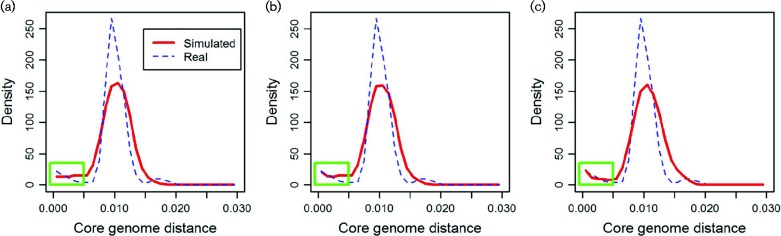
Effects of geographical sampling bias and a recent bottleneck on the core genome Hamming distance distribution. Strains from a simulated generation, representative of the average shape, were selected as the initial population (a). The green rectangle highlights the region of interest, showing the increase in the number of closely related strain pairs in the real data. (b) The distance distribution after taking a geographically structured sample, averaged over 20 independent replicates (red curve). (c) The effect of a population bottleneck, obtained by selecting a specified number of strains (here 100 out of 2000 strains in total) as possible ancestors from which the next generation was sampled with replacement. Bottlenecks of other sizes are shown in Fig. S10. The distribution for the real data is shown in each panel for comparison.

There are important ways in which our model does not capture the observed data; one such is the small peak in the distance distribution close to the origin. To determine what might produce this, we extended the model in two simple ways. (1) We created a geographically biased sample, reflecting the way the real data were collected, similar to previous work on relating genetic divergence to short transmission chains (Fraser *et al.*, 2005). (2) We examined the impact of a population bottleneck, acting as a collective proxy for processes whereby some strains leave more progeny than others, including recent selection (Fraser *et al.*, 2009). For example, a recent vaccine introduction has led to rapid changes in the prevalences of certain serotype groups in the population (Croucher *et al.*, 2013). Outputs from these extensions demonstrate that both mechanisms can contribute to the peak, whilst leaving the main mode in the distribution intact, and further work will focus on estimating their relative significance (Fig. 2). Another major feature contradicting the expectation is the separate mode in the upper right corner of the distance distribution corresponding to a sequence cluster (SC12) divergent from the main group. Animation S2 shows how such additional modes emerge with decreased recombination, suggesting limited exchange between SC12 and the rest of the population. Notably, the previously reported recombination rate for SC12, detecting recombination as anomalous tracts of SNPs in the alignment, is relatively high (Fig. S9). This suggests that SC12 may be recombining with strains unrepresented in the population or conceivably that the SNPs in question are the consequence of some selective process that means SC12 does not fit our model (additional results are presented in the supplementary text and Figs S6–S16).

## Conclusion

We imposed a soft limit on genome size by assuming in our model a small fitness penalty for increasing genome size beyond a given threshold (see Methods). An analogous assumption has also been used by others (Vogan & Higgs, 2011), and whilst some selective pressure against larger genomes likely exists, the approach seems overly simplified. The limit is needed for computational reasons, but it also accounts for the empirical observation that genome sizes are not constantly increasing. Importantly, the limit does not produce any heritable fitness differences between different combinations of genes and the results are robust over a wide range of possible parameter values. Previous models have approached the same issue by either letting genomes grow (Baumdicker *et al.*, 2012) or by coupling gene introductions and deletions (Haegeman & Weitz, 2012; Lobkovsky *et al.*, 2013), both of which also seem arbitrary. In reality, several explanations may underlie the observation. In our model, the assumption facilitates the fitting of the gene frequency histogram as a stationary condition, from which the dominant mode in the distance distribution follows, given sufficient shuffling of genes between strains by recombination. Surprisingly, no additional assumptions, such as niche adaptation or selection on individual genes, are needed to explain the mode. The equidistant sequence clusters predicted by the model are consistent with previous findings showing the majority of differences in gene content between strain clusters to be related to combinations of loci, rather than unique cluster-defining genes (Croucher *et al.*, 2014).

We have developed a parsimonious model of genome evolution and shown that it can capture important features of a bacterial population, including the distance distribution between the strains and the gene frequency histogram. In addition, we have used it to detect characteristics of data that are not concordant with neutral expectations. We have demonstrated the importance of recombination in producing the population structure, as represented by either the gene content or the core genome divergence. Despite several ways in which the model is idealized, it broadly estimates the population genetic parameters well. A remarkable fact is that the model predicts the population of equidistant strain clusters observed in the real data without recourse to selection or niche adaptation; however, we emphasize that our purpose here is not to reject selection, but merely to point out its redundancy in explaining this striking feature of the population structure. We used our model as a null hypothesis to detect features not expected by neutral processes. For example, closely related strains required an additional explanation, such as a bottleneck. Furthermore, strains that were more divergent than expected, forming a distinct mode in the distance distribution, may be rationally defined as distinct species. Thus, our model might serve as a definition for a ‘(preferably) genomically coherent’ species, which is an aspiration of systematicists in response to the growth of genomic data. Improved annotation of accessory genomes, coupled with extensions of our model, will enable us to ask whether the observed gene combinations are more or less frequent than we would expect to see by chance.

The model we have developed offers insights into the processes that generate genotypic clusters associated with species in recombinogenic bacteria (see also Shapiro & Polz, 2014). There are obvious similarities to the biological species concept in eukaryotes, in which sexual reproduction operates as a cohesive force preventing divergence of lineages (e.g. Higgs & Derrida, 1992). However, the differences between eukaryotic and prokaryotic reproduction make this a more general version of the concept that is also capable of considering recombination between things we might term species, without being sufficient to prevent those species clusters becoming distinct. The divergent cluster of ‘atypical pneumococci’ may be considered a separate species by our criteria, i.e. that it forms a distinct mode that cannot be explained by the mean recombination rate within the population. There are multiple mechanisms by which this could have occurred, but common to them all is insufficient recombination between the two clusters, allowing them to diverge. The cause of that barrier is impossible to determine from the present analysis, but could be intrinsic (the two clusters do not recombine efficiently) or ecological (isolates in the two clusters do not encounter each other often enough for recombination to efficiently shuffle their genomes). Further work is necessary to distinguish between these possibilities.

## Supplementary Data

715 Supplementary Data
