# Supplementary material for: Recombination produces coherent bacterial species clusters in both core and accessory genomes
Source: Microb Genom. 2015 Nov 5;1(5):e000038. doi: 10.1099/mgen.0.000038 (PMC5320679; doi:10.1099/mgen.0.000038)
Supplement: Supplementary file 1 — Supplementary Data [file mgen-01-38-s001.pdf]

Recombination produces coherent bacterial species clusters in both core and accessory genomes - Supplementary material

Pekka Marttinen, Nicholas J. Croucher, Michael U. Gutmann,
Jukka Corander, and William P. Hanage

Contents

1 Detailed Methods **2**
1.1 Model 2
1.2 Model fitting 3

2 Detailed Results **4**
2.1 Gene frequency histogram 4
2.2 Population structure 5
2.3 Sensitivity analyses 6

3 Supplementary Figures **7**

4 Supplementary Tables **17**

5 Supplementary Animation Captions **18**

1 Detailed Methods

1.1 Model

As the basis of our simulation, we use the Wright-Fisher model, in which the next generation of strains is sampled with replacement from the current generation. The model may be modified to incorporate fitness by sampling strains with unequal probabilities. Motivated by the biological observation that genome sizes are not constantly increasing, we include a multiplicative fitness penalty (using a factor 0.99) to an otherwise neutral model for each gene exceeding a pre-specified number of genes to prevent unrestricted growth.

In our model, we use two separate data structures to represent the genome of each strain: 1) gene content component, represented by a binary indicator vector showing the presence and absence of genes, and 2) gene sequence component, represented by a fixed number of gene sequences for which detailed evolution is simulated (Supplementary Figures 1A and 1B). The two components are present in the same strains, but otherwise their evolution is independent. In the gene content component we assume no fitness differences between genes, and, in particular, make no distinction between core and accessory genes. Results concerning gene content, for example the proportion of core and the gene frequency histogram, are derived from this component. Because one of our goals is to investigate the relationship between gene content and core sequence divergences (Figure 1), we include the gene sequence component into our model and use it to compute the core genome distances. For simplicity, we assume the same genes to be present in all strains in the gene sequence component.

The evolution of the gene content is driven by the following events, taking place between the sampling of strain generations with frequencies specified by the parameters of the model:

1. Introduction of a novel gene into the population
2. Deletion of a randomly selected gene from a randomly selected strain
3. Horizontal gene transfer between two strains in the population, resulting in the gene presence/absence status of the donor to be copied to the recipient (Supplementary Figure 1C).

For modeling the detailed evolution in the gene sequence component, we use the following events:

1. Mutation
2. Homologous recombination, in which an allele of the recipient is replaced by an allele from the donor (Supplementary Figure 1C).

A detailed parameterization of the model is shown in Tables S1 and S2. Our motivation for separating gene content and core sequence evolution stems from computational savings resulting from the fact that it is not necessary to simulate the detailed sequence evolution for all genes. As a preliminary experiment, we implemented also a model with the two components combined and similar results were obtained; however, fitting the model took considerably longer (results not shown).

Our model assumes that a gene can enter the population only once, after which its evolution is driven by drift and recombination. Furthermore, as observed with several real data sets, the potential of bacteria to recombine decreases with decreasing sequence similarity. Motivated by this, we make the same assumption as Fraser et al. (2007), and accept recombination proposals with probability that decreases exponentially with increasing sequence divergence using parameter values observed in real data sets (see Table S2 for details).

To further reduce computational complexity, gene sequences, whose detailed evolution is simulated, are represented using a low-dimensional feature space analogous to Fraser et al. (2007). In detail, each gene is represented by a vector of 10 integers, serving as abstract sequence features. Every time a mutation occurs in the gene, a randomly selected feature is incremented by one. The distance between two strains computed using the feature representation underestimates the real sequence distance due to an increased probability of two mutations occuring at the same location and we correct for the bias by mapping the estimated distances to the expected true distances, using a mapping obtained with Monte Carlo simulation (Supplementary Figure 2), which has an improved accuracy compared to an analytical correction used by Fraser et al. (2007) in the range of distances relevant for this study.

1.2 Model fitting

Our model has in total five free parameters: three governing the evolution of gene content (deletion rate, novel gene introduction rate, horizontal gene transfer rate) and two governing the detailed sequence evolution (mutation rate, homologous recombination rate). Given the complexity of the model, maximizing its likelihood is computationally infeasible. We employ a simulation-based inference method instead, which resembles the simulated method of moments (McFadden, 1989; Pakes and Pollard, 1989; Gourieroux and Monfort, 1997; Wood, 2010). The basic idea is to fit the model by matching summary statistics of the real data. A two-step algorithm is used for fitting the model:

- Fit the parameters for the gene content evolution using 1) the gene frequency distribution, and 2) the median clonality score (see below) over genes present in approximately half (40-60%) of the strains.
- Fit the parameters for the detailed sequence evolution using 1) the slope of the gene content vs. core genome distance relationship, and 2) the median linkage score (see below) over all core gene pairs.

Each optimization step consists of simulating multiple artificial data replicates over a set of values for parameters to be optimized, and measuring the similarity between the simulated and real data statistics using a similarity measure (see below). Due to simulation variability, the similarity score between the simulated and real data sets fluctuates even if exactly the same parameter values are used in different simulation runs. For this reason, we do not have a closed form formula for the relation between similarity score and parameters. We learn the relation for the range of plausible parameter values by non-parametric regression (Rasmussen, 2006). Our estimate is obtained as the parameter value that maximizes the learned regression function, which represents the expected similarity between the simulated and real data. Supplementary Figure 3 illustrates this procedure when learning the parameters for the detailed sequence evolution.

The model fitting procedure incorporates a subjective decision of selecting data summaries to use when matching the real and simulated data sets. Ideally, the summaries would identify the parameters unambiguously. When fitting the gene content component, for example, the gene frequency distribution statistic was found to be highly informative about deletion and novel gene introduction rates; however, it did not contain sufficient information for identifying the horizontal gene transfer rate. For learning the horizontal gene transfer and homologous recombination rates, we defined two additional data summaries, the clonality score and the linkage score, respectively. Each of these two scores was found to vary monotonically with the recombination rates, such that high rates indicated low clonality or linkage scores (Supplementary Figures 4 and 5). Details of the two scores are provided below.

The *clonality score* for a gene is defined on the basis of the fact that the gene divides the strains into two groups, those with the gene, and those without. In the absence of horizontal gene transfer, the two groups would correspond to different branches of a phylogenetic tree, and, consequently, the within-group strain distances would be expected to be smaller than the between-group distances. We define the clonality score as the quantile of within-group distances that corresponds to the 0.01st quantile of the between group distances. Thus, it measures the excess of closely related strains sharing the gene (or its absence) relative to the proportion of closely related strains with differing gene presence/absence status. The median score over all genes present in approximately half of the strains was used as the final summary as these are the most informative about recombination events (if a gene is very rare or common, the chance of seeing it donated is low). The *linkage score* for a pair of genes is defined as follows: the distances between the strains are calculated using sequences for each gene independently. The Spearman correlation of the distances between the genes is taken as the linkage score for the gene pair. In the absence of homologous recombination, the distances are expected to be highly similar, resulting in a high linkage score.

When fitting the gene content component, the similarity between real and simulated data was measured using

$$d_1 = -\log KL - \frac{1}{2s^2_{real}} \left(c_{simu} - c_{real}\right)^2 ,$$

where c_{real} is the median clonality score over genes having frequency 0.4-0.6 in the real data, s^2_{real} is the variance of the median clonality score obtained by bootstrapping, c_{simu} is the corresponding median

clonality score in the simulated data, and KL is the Kullback-Leibler divergence between the real and simulated frequency histograms. To account for sampling, the simulated histogram was computed by averaging over histograms for 30 bootstrap samples of 616 strains (the number of strains in the real data), sampled from the 2000 strains simulated. Before computing the KL-divergence, the gene frequency distributions were discretized into 7 bins using boundaries: (0, 0.02, 0.05, 0.2, 0.95, 0.98,1) and a single bin for genes with frequency exactly 1. Thus, the bins simultaneously captured all main characteristics of the frequency distribution: the proportion of the core genome, the slopes at each end of the histogram, and a bin to combine intermediate frequencies. When fitting the gene sequence component, the similarity between real and simulated data was measured using

$$d_2 = -\log((s_{simu} - s_{real})^2) - \log((l_{simu} - l_{real})^2),$$

where s_{simu} and s_{real} are the slopes of the distance distribution in the simulated and real data sets and l_{simu} and l_{real} are the median linkage scores between all core gene pairs in the simulated and real data sets.

2 Detailed Results

2.1 Gene frequency histogram

Results from a model fitted by matching the gene frequency histograms and the clonality scores between the real and simulated data sets are shown in Supplementary Figure 6. The histograms were obtained by running the model for 40,000 generations, and combining the results at a 1,000 generation interval after discarding the first 10,000 generations, which yielded approximately the same number of genes as observed in the real data. The figure shows a simulated histogram with the optimized parameter values and illustrates the impact of each parameter on the results.

The results show that the overall gene content distribution can be fitted accurately by modifying only three parameters: novel gene introduction rate, deletion rate and horizontal gene transfer (recombination) rate. Intuitively, increasing the rate at which novel genes are introduced in the population has a major impact on the proportion of genes present in a small proportion of strains. Furthermore, the deletion rate influences the ratio of the number of genes present in all strains (the core) and the number of genes present in almost all strains, as the former become the latter through deletions. Recombination rate has a minor impact on the gene frequency histogram. On the other hand, the clonality score increases from 0.046 with a high recombination rate to 0.52 with a low recombination rate, with the fitted rate yielding a clonality score equal to 0.11 (the value in the real data is 0.12).

The main visually detectable quantitative difference between the real data and the optimized model is that the proportion of genes with frequency between 98 and 100%, i.e., genes that are almost core, corresponding to the rightmost grey column, is slightly higher in the real data (6%) than in the optimized model (4%). The fit of this aspect could be improved by increasing the deletion rate; however, this would lead to an excess of other high-frequency genes (see the panel with high deletion rate in Supplementary Figure 6). One possible reason for the larger proportion of the 'almost core' genes in the real data is that some of them are actually core, but have not been annotated as such due to inconsistencies in the gene prediction algorithm's output.

As an example of a real data feature related to the gene content, not compatible with the model assumpations, rare genes (present in 2-4 strains) were typically found in closely related strains in the simulation, as a result of inheritance from a common ancestor, but not in the real data (Supplementary Figure 7). A detailed inspection revealed that many rare genes had originated through frameshift mutations (not included in the model) and the proportion of frameshifts among genes found in distant strains was significantly higher (58%) than among genes found in closely related strains (34%, p=3.7e-6). Note that our data were treated to remove likely false positive gene predictions (see Methods in the main text).

An important difference between our model and previous models is the inclusion of within-population HGT events, which we have shown to play a central role in generating the observed distribution of accessory loci. Another recent model has assumed genes can be donated from one strain to another (Baumdicker and Pfaffelhuber, 2013). The key difference in our new model is that HGT may lead not only to an acquisition, but also to a deletion of a gene, which is biologically motivated. This avoids the problem related to an excess of genes at intermediate to high frequencies not seen in real data

(Baumdicker and Pfaffelhuber, 2013), which follows when each HGT event increases the number of genes in the population. With our formulation, within-population HGT is not expected to change the gene frequency histogram, because the number of donor-recipient pairs resulting in a gene deletion is equal to the number of pairs leading to a gene acquisition. This explains the success of the previous models to explain the gene frequency distribution without HGT (Baumdicker et al., 2012; Collins and Higgs, 2012; Haegeman and Weitz, 2012; Lobkovsky et al., 2013). One consequence of our formulation is that rare genes are deleted with a higher frequency than commons genes, because the number of strains that can 'donate the absence' of the gene is higher. This is connected with recent results indicating that low frequency genes have different acquisition and deletion rates than other genes (Collins and Higgs, 2012); however, as discussed above, many rare genes seemingly transfer faster than expected even by our model.

2.2 Population structure

The fitted recombination rate resulted in a population structure with multiple strain clusters approximately equally distant from each other (Supplementary Figure 8), yielding the best match with the key features of the overall population structure observed in the real data, in which 16 sequence clusters were detected (Croucher et al., 2013). However, some aspects of the population structure were not accurately captured by the simple model. For example, the separate small mode in the top-right corner and the peak close to the origin in the heat map (Figure 1) seem not well accommodated by the fitted model, although the model assigned some probability mass to these regions also. The separate mode suggests that the corresponding sequence cluster 12 may have a lowered ability to recombine with the rest of the population. The peak close to the origin, on the other hand, means that strains in some sequence clusters are more closely related to each other than expected by neutral variation. A more detailed inspection of the real data revealed different sequence clusters to have different distance distributions, highlighting the fact that the fitted model was obtained by averaging over many independent evolutionary processes (Supplementary Figure 9).

In an attempt to gain a better understanding of mechanisms that could underlie the peak near the origin in the distance distribution, we experimented with two simple extensions of our model, compatible with the available background information (Croucher et al., 2013). In the first extension, a geographically structured sample was taken from the whole population, to account for the observed relatedness of strains from the same location and sequence cluster. We included each sampled strain multiple times following the joint distribution of sampling sites and sequence clusters in the real data, thus representing an upper bound on the effect achievable by the geographic structuring. In the second extension, a bottleneck was simulated on the population, acting as a simple proxy for other processes by which some strains produce more offspring than others, such as periodic selection and selective sweeps (Fraser et al., 2009). Example outputs from the extensions demonstrate the capability of both of them to explain some of the peak while leaving the main mode intact (Figure 2 and Supplementary Figure 10). However, we emphasize that the real data likely represent an outcome of many of the processes acting continuously and in conjunction, and with varying relative importances and timescales within different sequence clusters. Actually fitting the models, and selecting between them and more complicated alternatives would require explicit quantitative characterization of the differences between the sequence clusters, which is beyond the scope of this work.

The effect of recombination can be understood as follows: on one hand, it acts as a diversifying force for closely related strains, as the strains acquire recombinations from other distant strains. On the other hand, recombination prevents a strain from diverging further than the average strain distance by mixing genes between the strains. As a result of the two forces acting in opposite directions, the distance distribution ends up consisting of a single mode, clearly separate from the origin. Moreover, once recombination rate is sufficiently high for the mode to emerge, further increase does not change its location. This can be understood by noticing that recombination does not remove variation at any single locus, it only shuffles it across the strains. Because the strain distances are obtained as an average over distances at individual loci, it follows that after shuffling the different loci, the pairwise strain distances become concentrated around the mean locus-wise distance. The essentially same principles apply in the directions of both the axes, although details differ: in the direction of the x-axis (core genome distance), homologous recombinations are shuffling the variation in gene sequences caused by mutations. In the direction of the y-axis (gene content distance), the variation in gene content caused by deletions and introductions of novel genes is mixed between the strains by horizontal gene transfers.

By comparing the distance distributions between the three years in which the strains were sampled

(2001, 2004, 2007), one can test the conclusion that the mode in the distance distribution represents a stationary property of the population. Indeed, the distributions seems highly similar (Supplementary Figure 11), as expected on the basis of the results from the model; however, we note that mapping the time-scales between simulation and real data is not straightforward.

2.3 Sensitivity analyses

Our model does not assume separate core and accessory genomes, but the core emerges stochastically when genes become fixed. For comparison, we investigated assuming part of the core 'stable', i.e., deletion of these loci recuded fitness to zero, leaving no descendants. The results show that models with less than 30% of the core stable (of the whole core) could be fitted approximately equally well to the real data (Supplementary Figure 12). The fit decreased when the proportion of stable core was increased beyond 30%, when no parameter combination was able reproduce the frequency distribution adequately. In detail, the core ended up too large, and the proportion of common accessory genes with frequency between 50% to 100% too small when many stable core genes were assumed (Supplementary Figure 13). This result is in contrast with a recent estimate that genes in the stable category would account for approximately 80% of the core genome, which was obtained by fitting a model assuming one stable (essential) and two accessory gene categories to the frequency histogram (Collins and Higgs, 2012); however, the histogram was based on 14 *S. pneumoniae* genomes only.

We included in our model a multiplicative fitness penalty, equal to 0.99, for each gene beyond a pre-specified limit for the number of genes. Changing this parameter in the range from 0.95 to 0.999 does not affect the gene frequency histogram (Supplementary Figure 14), and, consequently, also the location of the mode in the gene content Jaccard distance is unaffected. The only measurable difference was that the average genome size increased from 0.95 to 1.02 relative to the limit for the genome size, and development of further summaries is required to formally fit the parameter. In the analyses the number of strains in the population was equal to 2000. Neither decreasing this to 1000 nor increasing to 4000 affects the main conclusions (Supplementary Figures 15 and 16). The only notable difference is a minor decrease in the overall variation in the population over time with respect to increasing population size, as expected, resulting in smaller variance in the summaries.

References

Baumdicker, F., W. R. Hess, and P. Pfaffelhuber. 2012. The infinitely many genes model for the distributed genome of bacteria. Genome Biology and Evolution 4:443–456.

Baumdicker, F., and P. Pfaffelhuber. 2013. The infinitely many genes model with horizontal gene transfer. arXiv preprint arXiv:1301.6547 .

Collins, R. E., and P. G. Higgs. 2012. Testing the infinitely many genes model for the evolution of the bacterial core genome and pangenome. Molecular Biology and Evolution 29:3413–3425.

Croucher, N. J., J. A. Finkelstein, S. I. Pelton, P. K. Mitchell, G. M. Lee, J. Parkhill, S. D. Bentley, W. P. Hanage, and M. Lipsitch. 2013. Population genomics of post-vaccine changes in pneumococcal epidemiology. Nature Genetics 45:656–663.

Fraser, C., E. J. Alm, M. F. Polz, B. G. Spratt, and W. P. Hanage. 2009. The bacterial species challenge: making sense of genetic and ecological diversity. Science 323:741–746.

Fraser, C., W. P. Hanage, and B. G. Spratt. 2007. Recombination and the nature of bacterial speciation. Science 315:476–480.

Gourieroux, C., and A. Monfort. 1997. Simulation-based econometric methods. Oxford University Press.

Haegeman, B., and J. S. Weitz. 2012. A neutral theory of genome evolution and the frequency distribution of genes. BMC Genomics 13:196.

Lobkovsky, A. E., Y. I. Wolf, and E. V. Koonin. 2013. Gene frequency distributions reject a neutral model of genome evolution. Genome Biology and Evolution 5:233–242.

McFadden, D. 1989. A method of simulated moments for estimation of discrete response models without numerical integration. Econometrica 57:995–1026.

Pakes, A., and D. Pollard. 1989. Simulation and the asymptotics of optimization estimators. Econometrica 57:1027–1057.

Rasmussen, C. E. 2006. Gaussian processes for machine learning. Citeseer.

Wood, S. N. 2010. Statistical inference for noisy nonlinear ecological dynamic systems. Nature 466:1102–1104.

3 Supplementary Figures

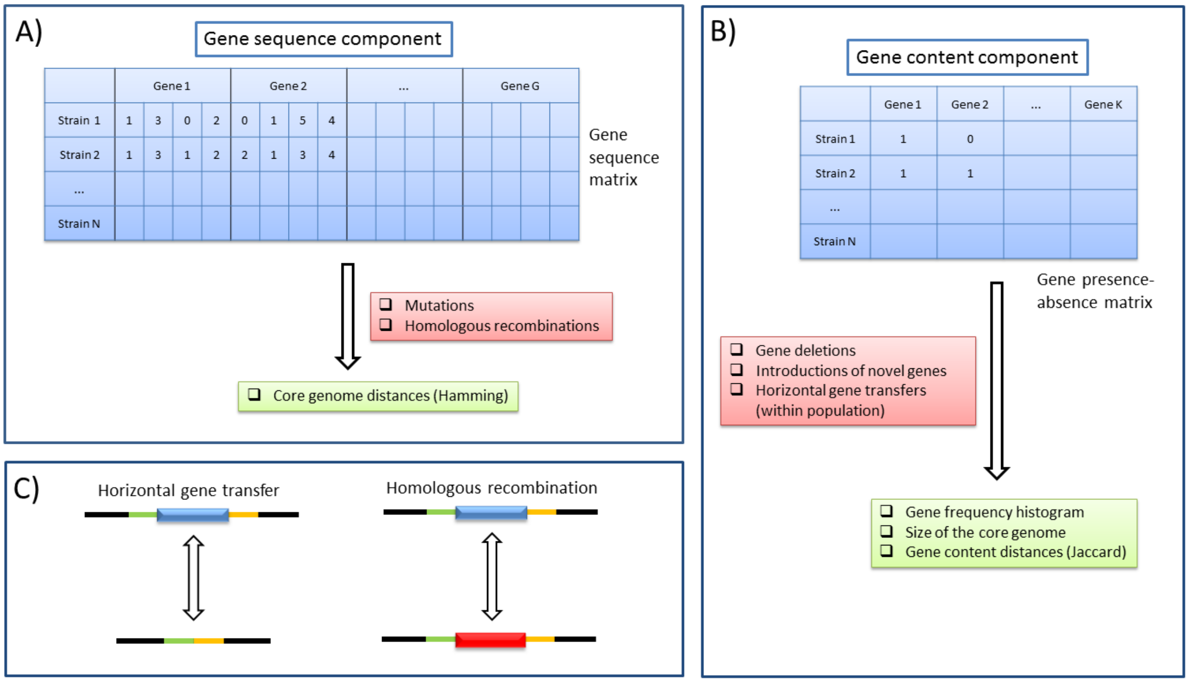

Figure 1: Schematic illustration of the model. A/B) Gene sequence/gene content components of the model. The related data structures are colored blue, the evolutionary forces acting on the components red, and the outputs derived from the components green. C) Recombination events implemented in the model. In a horizontal gene transfer, a sequence encompassing a gene (blue rectangle) may replace a sequence in another strain without the gene, or *vice versa*. In a homologous recombination an allele (blue rectangle) is replaced by another allele (red rectangle) of the same gene, or *vice versa*.

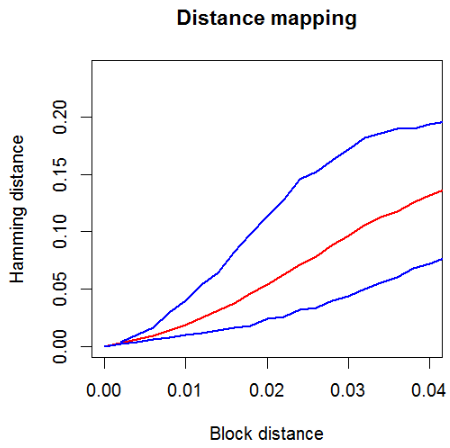

Figure 2: Mapping of sequence distances from low-dimensional sequence representation to Hamming distances resulting from the same number of mutations following the Jukes-Cantor model. The mapping was derived from results obtained by simulating the low-dimensional and the full model in parallel multiple times. The red line shows the mean of the distribution, blue lines the 5th and 95th percentiles.

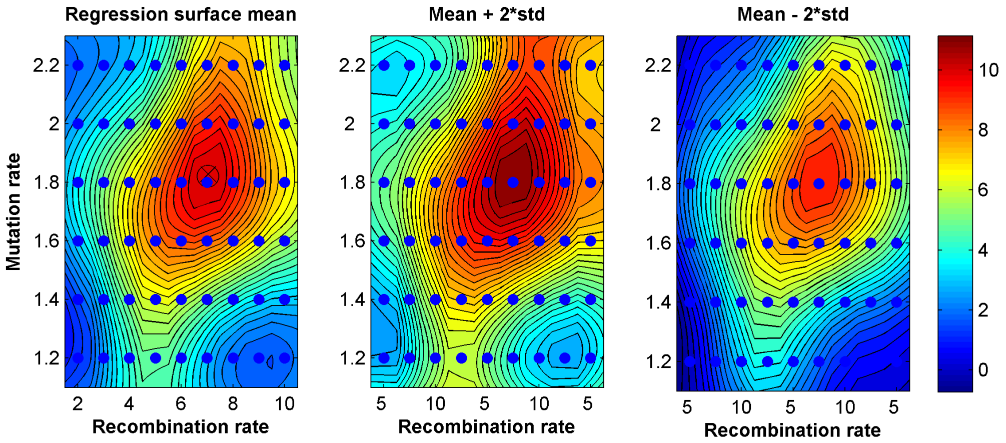

Figure 3: A distribution for the similarity scores between the simulated and real data. The similarity is computed using formula $-\log((s_{simu} - s_{real})^2) - \log((l_{simu} - l_{real})^2)$, where s_x refers to the slope of the gene content vs. core genome distance distribution, l_x to the median linkage score over all core gene pairs, and the subscript x specifies whether real or simulated data is in question. The blue dots show the parameter combinations at which the simulations were run and the cross denotes the location of the optimal parameter value.

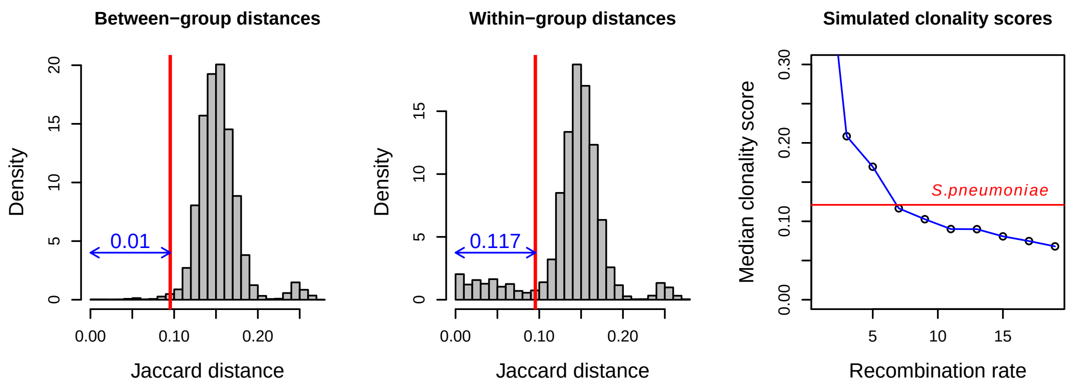

Figure 4: Illustration of the clonality scores. A gene selected randomly from the real data divides the strains into two groups, those with and without the gene. The first and second panels show the between-group and within-group gene content (Jaccard) strain distances. The quantile of the within-group distances corresponding to the 0.01st quantile of the between-group distances is defined as the clonality score of the gene (here 0.117). The last panel shows how the median clonality score (computed over genes with frequency 40-60%) varies in a simulation as a function of horizontal gene transfer rate.

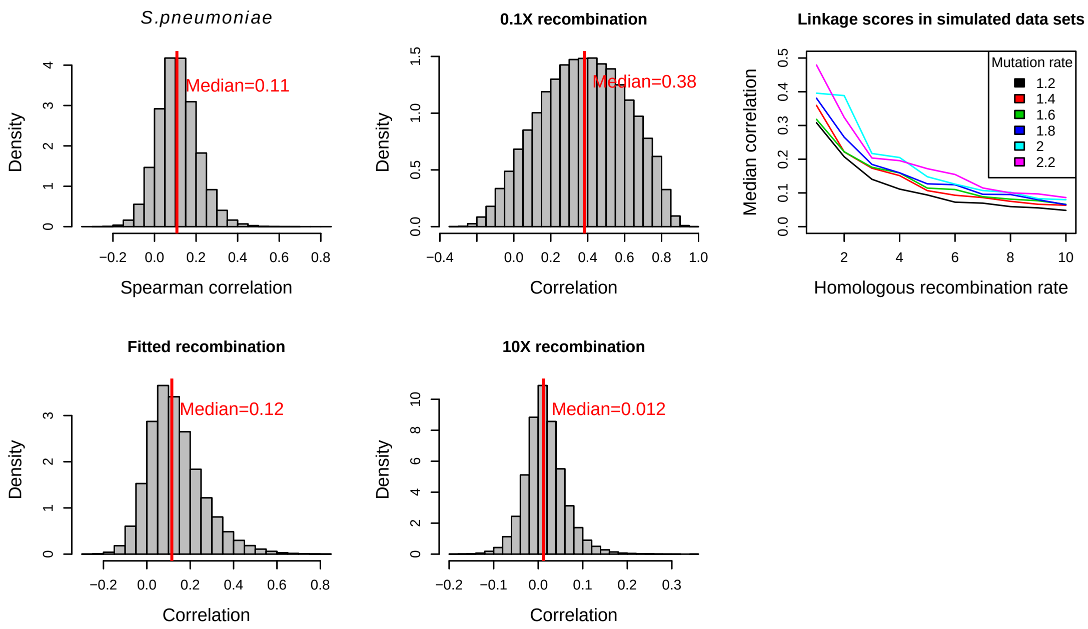

Figure 5: Illustration of the linkage scores. A linkage score for a pair of core genes is defined as the Spearman correlation of strain Hamming distances computed separately at the two genes. The panels show the distribution of linkage scores in the real data, and in simulated data sets with fitted/decreased/increased homologous recombination rates. The panel on the right shows how the median linkage score over all core gene pairs varies in the simulations as a function of the homologous recombination and mutation rates. To investigate the sensitivity of the median score to different levels of variation observed in different genes in the real data, we re-computed the median score after removing all genes with less than 16 or 27 SNPs (10th and 20th percentiles of the SNP count distribution). The median score changed from 0.108 to 0.112 to 0.116, indicating a negligible impact on the recombination rate estimate.

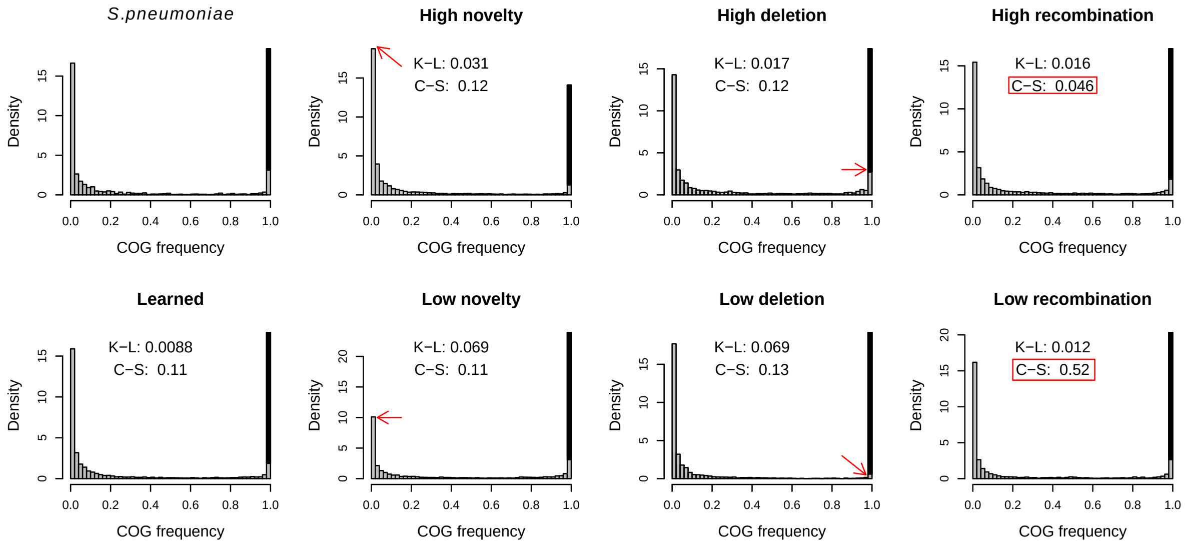

Figure 6: Gene frequency distributions. The x-axis shows the proportion of strains in which a gene is present, such that rare genes appear on the left, and common genes on the right. The black column represents genes present in all strains, *i.e.*, the core genome. Individual panels, in columnwise order, show results for the *S. pneumoniae* data, simulated data with fitted parameter values, simulated data with increased/decreased novel gene introduction rates, simulated data with increased/decreased deletion rates, and simulated data with increased/decreased homologous recombination rates. Kullback-Leibler (K-L) divergence between the real and simulated histograms, and the clonality score (C-S) are shown for the simulated results (the clonality score for the real data was approximately 0.12). The read arrows and boxes highlight features most affected by the respective parameters.

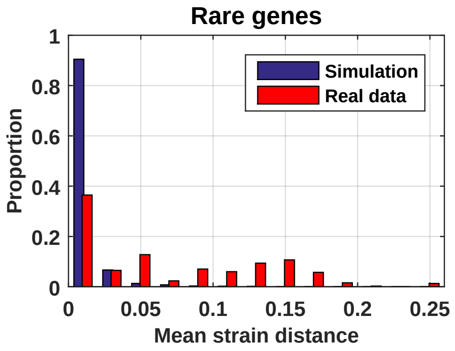

Figure 7: Mean gene content (Jaccard) distances between strains sharing a rare gene. The results show that strains sharing a rare gene are usually closely related to each other in a simulation (blue). In the *S. pneumoniae* data, a relatively large proportion of rare genes are found in distant strains (red).

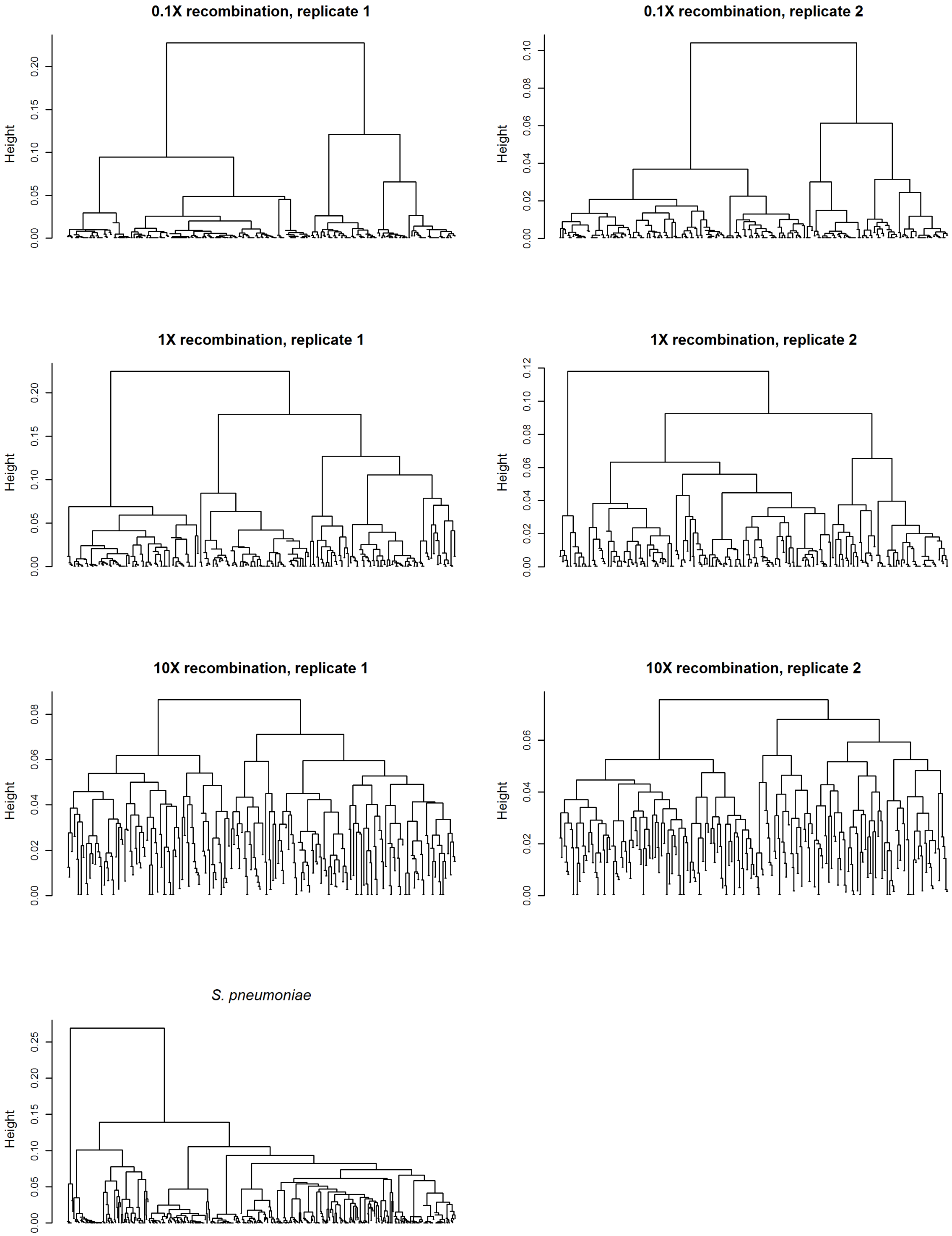

Figure 8: Simulated phylogenetic trees. The trees are based on core Hamming distances, and estimated using the simple complete linkage hierarchical clustering. Results for the fitted (1X), decreased (0.1X), and increased (10X) recombination rates are shown, along with the *S. pneumoniae* data for reference. The long branch in the tree for the real data separates strains in the divergent sequence cluster 12 from other strains. The characteristics of the tree with the decreased rate include dense clusters in the ends of long branches. On the other hand, the increased rate corresponds to a tree with star-tree like characteristics, with none of the strains very close or distant from the other strains. The fitted rate results in a compromise between the two extremes.

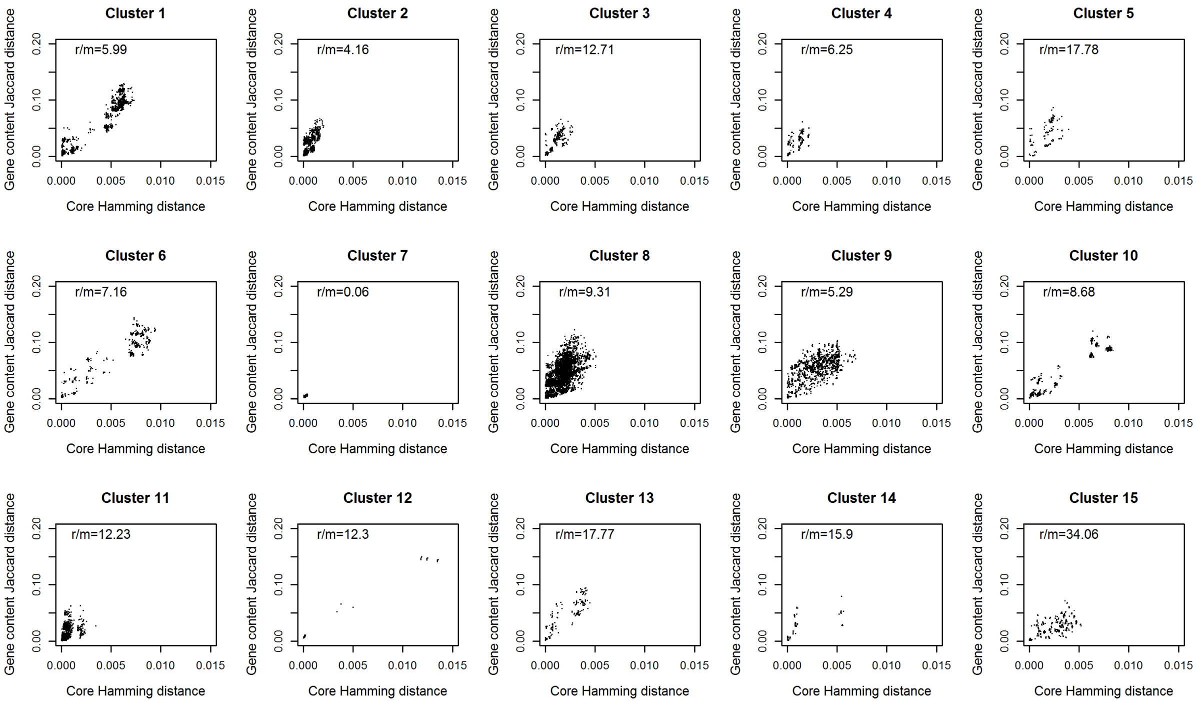

Figure 9: Gene content Jaccard distances vs. core genome Hamming distances for strains within different sequence clusters in the *S. pneumoniae* data set. The r/m values in the panels show estimates for the numbers of substitutions introduced by recombinations vs. mutations in the sequence clusters, and are taken from Croucher *et al.* (2013). No apparent relation between r/m and the shape of the distribution seems to exist.

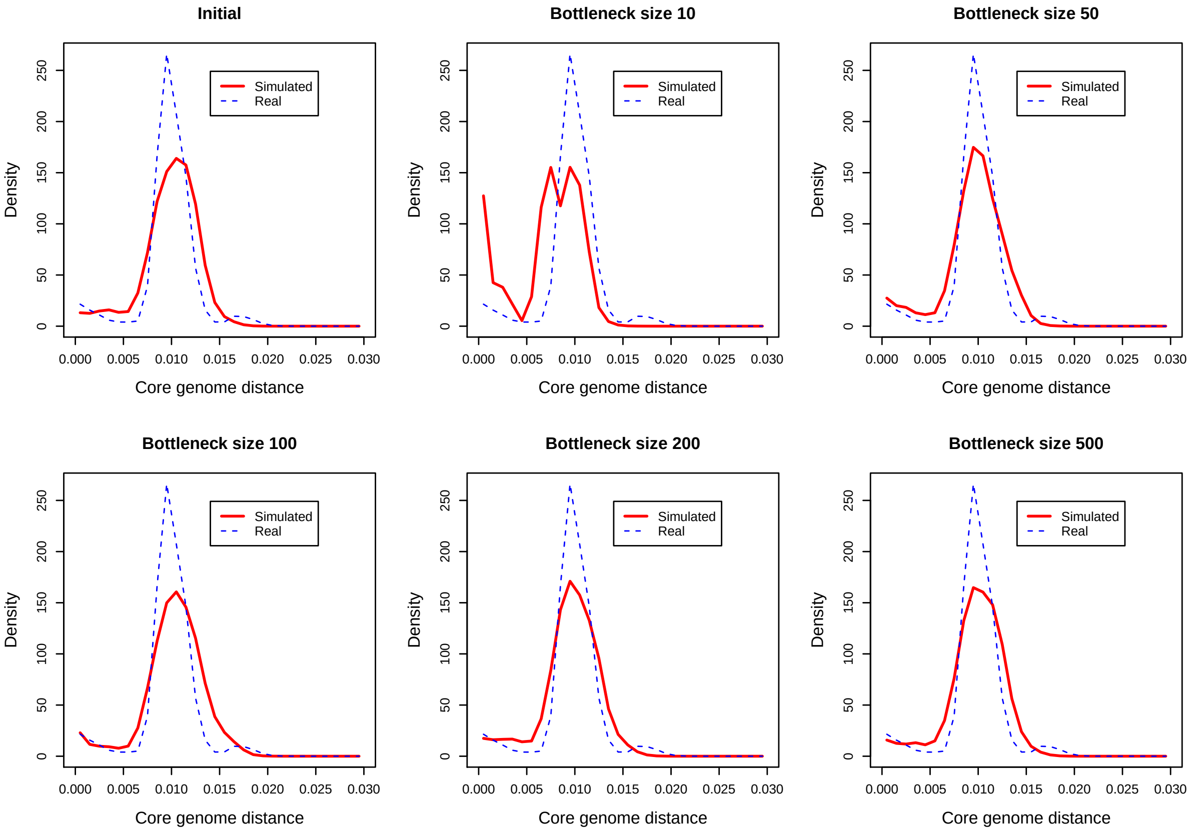

Figure 10: Effect of a population bottleneck on the core genome Hamming distance distribution. Strains from a simulated generation, representative of the average shape, were selected as the initial population. A bottleneck was simulated by selecting a specified number of strains (out of 2,000 strains in total) as possible ancestors from which the next generation was sampled with replacement. The bottleneck with size 100 seems to produce the most similar peak near the origin to the one observed in the real data.

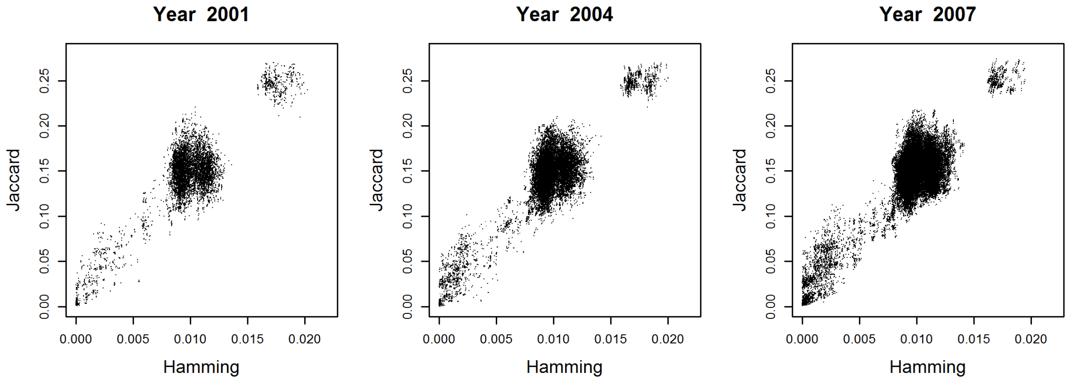

Figure 11: Gene content Jaccard distances vs. core genome Hamming distances for strains sampled in different years in the *S. pneumoniae* data set. The numbers of isolates in the different years were: 133 (2001), 203 (2004), and 280 (2007).

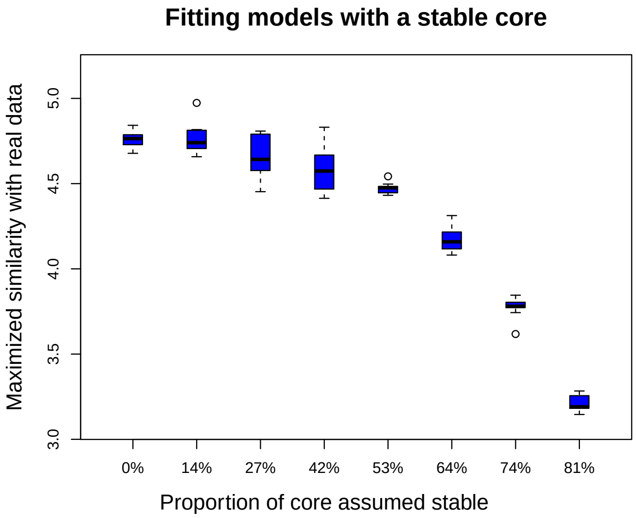

Figure 12: Maximized similarity scores between simulated and real data for different proportions of stable core genome out of the whole core genome. Different numbers of stable core genes were assumed, and the model was optimized 10 times independently. The boxplots show the similarity scores between the real data and the optimized models in the 10 optimization rounds.

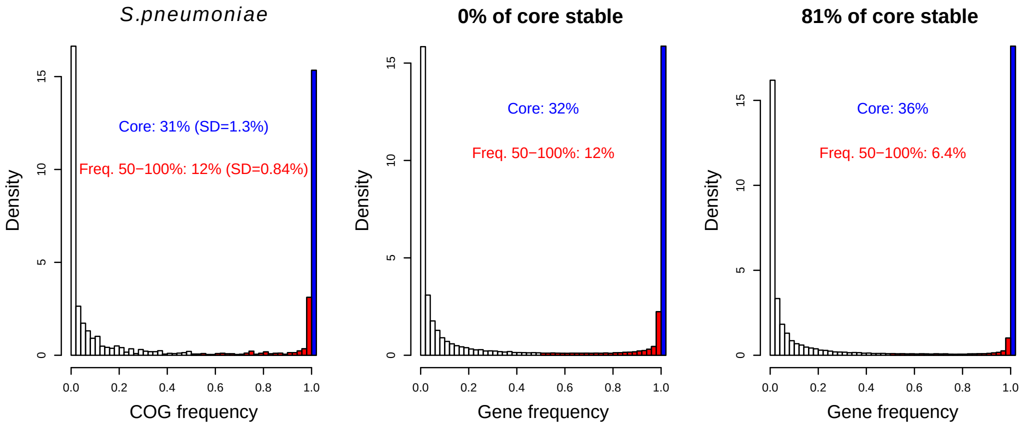

Figure 13: Effect of assuming a stable core on the gene frequency histogram. The left-most histogram shows for reference the frequencies in the real data. The other two histograms show fitted histograms, averaged over 10 optimization rounds, from two different models, one assuming no stable core, the other assuming that on average 81 per cent of core (out of the whole core) is stable. Additional annotation in each panel specifies: the proportion of genes that are present in all strains, i.e. the core (blue), proportion of non-core genes that are present in 50-100 per cent of the strains (red).

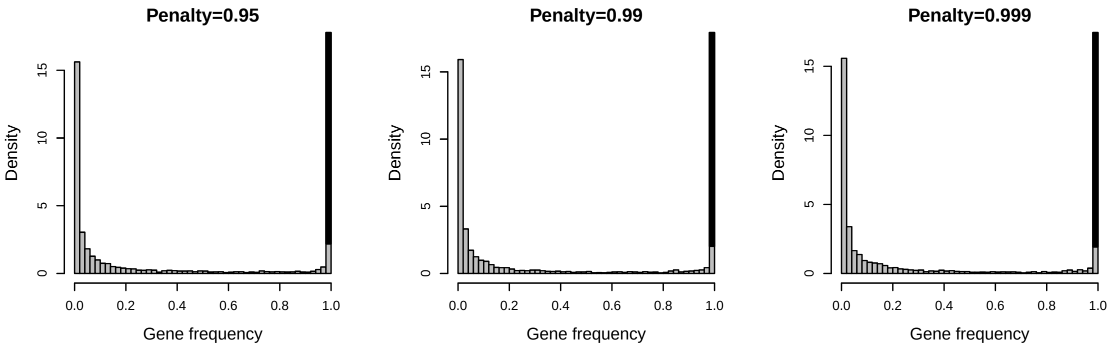

Figure 14: Gene frequency histograms when the fitness penalty for increasing the number of genes is changed in the range from 0.95 to 0.999.

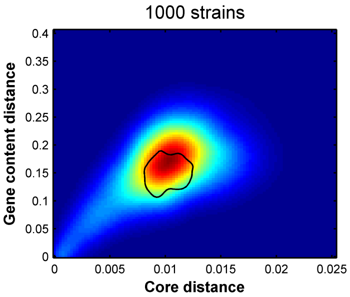

Figure 15: Impact of the number of simulated strains on the distance distributions.

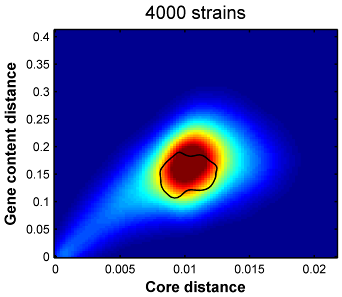

Figure 16: Impact of the number of simulated strains on the gene frequency histograms.

4 Supplementary Tables

**Parameter**	**Description**	**Fitted value**
deletion.rate	Mean number of gene deletions per generation (relative to the size of the core genome).	0.066
novel.gene.introduction.rate	Mean number of introductions of novel genes per generation (relative to the size of the core genome).	0.18
horizontal.gene.transfer.rate	Mean number of horizontal gene transfer attempts per generation per gene.	7.4
mutation.rate	Mean number of mutations per generation per gene (gene sequence component).	1.8
homologous.recombination.rate	Mean number of homologous recombination event attempts per generation per gene (gene sequence component).	7.0

Table 1: Evolutionary parameters in the model.

**Parameter**	**Description**	**Value**
num.strains	Number of sequences simulated.	2000
sequence.component.size	Number of genes for which detailed evolution is simulated in the gene sequence component.	40
genome.size	Number of genes that can be present in the gene content component in a strain without fitness cost.	60
fitness.cost.per.gene	Fitness cost per excess gene applied to strains which have the number of genes larger than *genome.size*	0.99
rec.acceptance.par	A recombination attempt is accepted with probability 10^{-Ax}, where A is *rec.acceptance.par*, and x is the local sequence divergence calculated over the gene affected by the recombination (homologous recombination) or, when the local divergence is not available in the horizontal gene transfers (full sequences not simulated), the overall Jaccard distance between the donor and the recipient (mapped to the corresponding Hamming distance).	18
gene.length	The length in base pairs of a gene for which detailed evolution is simulated	500

Table 2: Simulation meta-parameters

5 Supplementary Animation Captions

Animation 1: Evolution of the strain distance distribution with fitted parameter values. The simulation was run for 25,000 generations and the animation was created by plotting the distance distribution at 100 generation interval. Fig. 1e of the main text was created by averaging over the generations, after discarding the first 10,000 generations. (*animation_fitted_recombination.avi*)

Animation 2: Evolution of the strain distance distribution with between strain recombination rate multiplied by a factor of 1/10. (*animation_0.1X_recombination.avi*)

Animation 3: Evolution of the strain distance distribution with between strain recombination rate multiplied by a factor of 10. (*animation_10X_recombination.avi*)
